# Hospital Notes

**Published:** 1871-06

**Authors:** C. C. F. Gay

**Affiliations:** Surgeon to the Buffalo General Hospital


ART. II.—*Hospital Notes.* By. C. C. F. Gay, M. D., Surgeon to the Buffalo General Hospital. *Operation for the Radical Cure of Hydrocele.*

———

Case 1st.—Mr. M—, aged 22 years, from Rochester, entered hospital March 14th, having hydrocele upon right side; had been tapped once, and again treated for radical cure by injection of iodine fluid; had now re-accumulated until the hydrocele had attained a very larg size. On the 15th I introduced trocar and drew off half a pint of straw colored fluid, afterwards inserted a silk seton, consisting of a double silk threat. The seton was introduced by a needle four inches in length, having an eye at its point, the same needle I use for the radical cure of hernia. The needle entered the sac at the fundus of the scrotum, and was made to emerge at a point three inches above the point of entrance. This seton is expected to excite sufficient inflammation to effect a radical cure. In three days from date of operation the sac filled up again, which necessitated the use of the trocar on account of pain from over distention. The next day the patient removed the seton on his own motion, because, as he said, "he could no longer stand it." There has been considerable pain and swelling of the scrotum and testis, but these gradually subsided, and the swelling and enlarged gland are diminishing, and he feels so comfortable that in ten days after operation, he left hospital for his home.

April 20th.—He visited the hospital. There is no re-accumulation of fluid, the testis is nearly reduced to its normal size, and the patient feels confident of radical cure.

Case 2.—Mr. B—, aged 44 years, has hydrocele on left side, small. Has been cured, he says, of a hydrocele upon right side. The gland feels hard, does not fluctuate, and the diagnosis is so doubtful that I first made use of the exploring needle. On the 15th, I operated as in the former case, using the seton after the trocar, passing the needle through the puncture made by this instrument. The point of entrance and exit were two and a half inches removed from each other. In this case I determined to make trial of the tolerance of the seton to ascertain how long it might be allowed to remain with impunity. I therefore, with this view, ordered anodynes to allay pain, and kept the seton in situe thirty days. The parts became much enlarged, an abscess formed, which was opened, giving free escape to pus, when the swelling was at once measurably reduced, the patient leaving the hospital before the testicle had resumed its normal shape, but so much diminished in size as to cause no longer any discomfort.

Case 3d.—This hydrocele was upon the right side, the seton was first introduced, and immediately afterwards the fluid was drawn off with the trocar. The seton was removed on the fifth day. Its presence was attended with considerable pain and inflammation, but the cure was complete in a fortnight. This operation was upon a private patient.

REMARKS.—Of the several operations for the radical cure of this affection, the one here employed and described, perhaps, presents as many advantages and as few objections as any other. It is certainly as safe and as easy of execution as any, and I know of no reason why it is not as efficient as any. It is attended with as little pain, both during and after the operation, as any of the various methods now employed. If sufficient number of cases should prove the operation inefficient, then there might be added to it or employed along with it, the injection of iodine. Before the cannula is removed a drachm of this tincture might be thrown into the sac through the cannula. If any advantage could possibly result in this double method it would arise from the fact of the more speedy excitation of the inflammatory process. I think the better way of operating by this method is to insert the seton first and immediately thereafter draw off the water with the trocar; but the fluid would escape even without the employment of the trocar, it will dribble away, and in time escape, but the objection arises, to the non-employment of the trocar, which will be at once anticipated, viz., the delay of the inflammatory process.

Two or three days is, doubtless, sufficient length of time for the seton to remain, since inflammation will have been attained in degree and amount to affect the closure of the sac.

In an operation made May 5th, 1871, upon a female patient in the country, I thrust the needle, which is four inches in length, through the scrotum its entire length, thus making the point of entrance and exit nearly or quite four inches apart; fluid dribbled away from both the lower and upper punctures, but I did not leave the fluid to escape in this way, nor deem it best to so leave it. I made use of the trocar, and the case terminated favorably, although the patient was somewhat infirm from previous disease and advanced age. Very little pain attended the operation or followed it, and there was no constitutional disturbance. Whether this method of operating for hydrocele is to take precedence over that of swabbing out the sac with iodine, or the other operation of laying open the sac entirely and packing it with lint, thus allowing the healing process to commence from below and within, remains an open and moot question. I have reported the cases in the order in which they occurred with a view to assist in solving the question, and if possible to substitute, for an operation somewhat dangerous, quite painful and protracted in its healing processes, one comparatively painless, simple of execution, little dangerous, and with duration of after treatment much abridged.

